# Toxin-producing *Escherichia coli*: a long-term retrospective study in dogs and cats between 2017 and 2023 in Italy

**DOI:** 10.3389/fvets.2025.1557445

**Published:** 2025-05-12

**Authors:** Giovanna De Luca, Giorgia Costantini, Luca Borrelli, Pasquale Izzo, Nunzia Riccone, Francesca Del Piano, Ornella Valvini, Amalia Gallo, Clementina Auriemma, Flora Alfano, Marco Paone, Giovanni Sgroi, Peyman Khademi, Nicola D’Alessio, Giovanna Fusco, Maria Gabriella Lucibelli

**Affiliations:** ^1^Experimental Zooprophylactic Institute of Southern Italy, Naples, Italy; ^2^Department of Veterinary Medicine and Animal Production, University of Naples Federico II, Naples, Italy; ^3^Department of Microbiology and Food Hygiene, Faculty of Veterinary Medicine, Lorestan University, Khorramabad, Iran

**Keywords:** cat, dog, *Escherichia coli*, Italy, public health, toxin

## Abstract

**Introduction:**

Toxin-producing *Escherichia coli* are gastrointestinal agents found in both animals and humans, potentially leading to mild-to-severe pathogenic outcomes. Therefore, this study aimed to assess the prevalence of toxin-producing *E. coli* in owned and stray dogs and cats in Southern Italy in order to provide insights into the epidemiology of these zoonotic bacterial infections.

**Methods:**

During necropsy, organ swabs (i.e., intestine, liver, lung, spleen, lymph node, and brain) from dogs and cats were collected and analyzed to isolate *E. coli* colonies through bacterial culture between 2017 and 2023. The isolated strains were then subjected to biomolecular investigation for pathogenicity factors.

**Results:**

Out of 911 animals, 451 (49.5%) tested positive for *E. coli*, including 252 (56.1%) dogs and 199 (43.1%) cats. The higher prevalence among dogs was statistical significant (*p* < 0.01) and associated with a higher risk of infection (*OR* = 1.69). However, no statistically significant difference in prevalence over the years was found (*p* = 0.150). At least one virulence factor was detected in 22% of animals, with 12% exhibiting pathogenicity factors (CNF, CDT, LT, and ST) and 10% showing virulence genes (vtx1, vtx2, and eae). Cats were significantly more likely to produce verocytotoxin compared to dogs (*p* = 0.020, *OR* = 2.04).

**Discussion:**

These findings suggest a wide circulation of toxin-producing *E. coli* in dogs and cats in Southern Italy, highlighting the importance of routine screening for these agents to ensure animal welfare and public health.

## 1 Introduction

*Escherichia coli* is a Gram-negative bacterium commonly found in the intestines of various species, including humans, dogs, and cats (1).

A wide variety of animal species, particularly cattle, serve as reservoirs of *E. coli* infections, hosting the microorganisms in their large intestines. Thus, animal-derived food products represent one of the primary sources of infection in humans (2).

Although the majority of *E. coli* strains are part of the normal gut flora and typically do not cause health issues (3), some strains can acquire virulence factors that make them pathogenic, enabling them to invade extraintestinal sites (4). These pathogenic strains of *E. coli* can be classified into several pathotypes: enterotoxigenic *E. coli* (ETEC), enteropathogenic *E. coli* (EPEC), verocytotoxin-producing *E. coli* (VTEC), and necrotoxigenic *E. coli* (NTEC), all of which are associated with infections in both animals and humans (5). NTEC strains are known for producing a cytotoxic necrotizing factor (CNF), which induces significant damage to host cells. CNF influences regulatory factors of the cellular cytoskeleton, triggering a cascade of events leading to cellular dysfunction and death (6). NTEC have been isolated from animals and humans, suggesting possible zoonotic transmission (7, 8).

The pathogenicity of VTEC strains is closely related to the production of exotoxins called verocytotoxin (vtx), a family of Shiga-like toxins causing haemorrhagic colitis and hemolytic uremic syndrome (HUS) (9). For instance, individual VTEC strains produce one or both toxins—vtx1 and vtx2—with the latter being associated with more severe disease forms (9). Enterotoxigenic *E. coli* (ETEC) is one of the leading causes of traveler’s diarrhea. Its virulence is characterized by the presence of specific adhesins and enterotoxins. ETEC toxins are divided into two groups: heat-stable enterotoxins (STI, STII) and heat-labile enterotoxins (LTI, LTII), both of which are associated with watery diarrhea and gastrointestinal illness (10).

Enteropathogenic *E. coli* (EPEC) is known to cause characteristic lesions, named *attaching and effacing* (A/E) lesions, which result in the effacement of microvilli and the formation of actin pedestals (11–13).

Therefore, this study aimed to investigate the prevalence of toxin-producing *E. coli* in dogs and cats in Southern Italy between 2017 and 2023, in order to assess the circulation of these agents and their potential health implications for animals and humans.

## 2 Materials and methods

### 2.1 Study area and sampling

From January 2017 to December 2023, veterinary practitioners in the Calabria and Campania regions (Southern Italy) collected carcasses of owned and stray dogs and cats after road accidents or apparent natural causes in accordance with National Laws (D.lgs. no. 194/2008 and 32/2021) by the Italian Ministry of Health were collected. All carcasses were delivered to the Animal Health Department of the Experimental Zooprophylactic Institute of Southern Italy (Naples, Italy) for a complete necropsy examination. The carcasses were dissected under sterile conditions, and cotton swabs were collected after cauterization with a spatula on the surface of the organ. Swabs were performed on organs displaying characteristic lesions, or in their absence, on target organs such as the intestine, liver, spleen, lung, and brain.

### 2.2 Cultures

For bacteriological examination, all the organ swabs were analyzed under a laminar flow hood on a plate of no-salt Agar McConkey (MCSS) and a plate of Tryptone Soya Agar +5% Blood (TSA), which were incubated aerobically at 37°C for 24 h. For intestinal swabs, sowing was carried out after preparing graduated dilutions in the ratio 1:10 in sterile peptone saline solution up to dilution 10^4^ to obtain isolated colonies on the culture medium. The diluted solution (10 μL) was transferred to a sterile loop and sown on the solid medium, aerobically incubated at 37°C. After 24 h, Gram staining, as well as catalase and oxidase tests, were performed on both usual and questionable colonies. The suspicious colonies obtained by Gram staining and catalase/oxidase tests were biochemically confirmed at the genus/species level using the Vitek^®^ 2 COMPACT automated system (Biomérieux, Marcy-l’Étoile, France). All *E. coli* colonies that tested positive for biochemical analysis were then subjected to molecular characterization to identify the presence of any genes encoding pathogenic factors.

### 2.3 DNA extraction and quantification

DNA was extracted from each *E. coli* strain using a boiling protocol (14) in which a single colony was suspended in 200 μL of water at 100°C for 10 min and pelleted with cellular debris. To assess the analytical sensitivity of the PCR assays, the DNA concentration of samples was determined by biophotometry BioPhotometer plus (Eppendorf AG, Hamburg, Germany).

### 2.4 PCR protocols

Virulence genes (i.e., vtx1, vtx2, and eae) were assessed by multiplex PCR in a total volume of 40 μL containing 25 μL HotStarTaq DNA Polymerase, 1 μL of each primer, 9 μL of Q solution (Qiagen, Hilden, Germany), and 10 μL of DNA template. The primer concentration was 0.2 μM, and the amplification conditions included an initial denaturation at 95°C for 15 min, followed by 35 cycles of 1 min of denaturation at 95°C, 2 min of annealing at 65°C for the first 10 cycles, decrementing to 60°C by cycle 15, and 1.5 min of elongation at 72°C incrementing to 2.5 min from cycles 25 to 35.

For CDT and CNF, an end-point PCR was run in a total volume of 50 μL containing 25 μL of Master mix Promega, 3 μL of each primer, and 2 μL of DNA template. The primer concentration was 0.6 μM, and amplification conditions included an initial denaturation step at 94°C for 2 min, followed by 30 cycles, consisting of 30 s of denaturation at 94°C, 1 min of annealing at 52°C, and 2 min of elongation at 72°C, with a final extension step of 5 min at 72°C.

For LT and ST, an end-point PCR was run in a total volume of 25 μL containing 12.5 μL of HotStarTaq DNA Polymerase (Qiagen, Hilden, Germany), 1.25 μL of each primer, and 3 μL of DNA template. The primer concentration was 0.5 μM, and the amplification conditions included an initial denaturation step at 95°C for 15 min, followed by 35 cycles of 1.5 min of denaturation at 94°C, 1.5 min of annealing at 60°C, and 1.5 min of elongation at 72°C, with a final extension step of 7 min at 72°C. The amplicons obtained were visualized by automated capillary electrophoresis with QIAxcel instrument (Qiagen, Hilden, Germany).

All details of the primers used in this study are listed in Table 1 (15–19).

**Table 1:** Primer sets used in this study for detecting pathogenicity factors and virulence genes.

Primer name	Primer sequence (5′-3′)	Target gene	PCR product (bp)	Reference
STX1 F	ATAAATCGCCATTCGTTGACTAC	stx1/vtx1	180	(15)
STX1R	AGAACGCCCACTGAGATCATC			
STX2 F	GGCACTGTCTGAAACTGCTCC	stx2/vtx2	255	(15)
STX2 R	TCGCCAGTTATCTGACATTCTG			
EAE F	GACCCGGCACAAGCATAAGC	eae (intimine)	384	(15)
EAE R	CCACCTGCAGCAACAAGAGG			
CNF F	CAATGGCAACAAAAATACCTTC	Cytotoxic necrotizing factor (CNF)	1,111	(6)
CNF R	GAACGACGTTCTTCATAAGTATC			
CDT F	GAAAATAAATGGAATATAAATGTCCG	Cytolethal distending toxin (CDT)	499	(16)
CDT R	AAATCACCAAGAATCATCCAGTTA			(17)
LT F	AGCAGGTTTCCCACCGGATCACCA	Heat-labile entero toxin (LT)	132	(18)
LT R	GTGCTCAGATTCTGGGTCTC			
ST F	GCTAATGTTGGCAATTTTTATTTCTGTA	Heat–stable enterotoxin (ST)	190	(19)
ST R	AGGATTACAACAAAGTTCACAGCAGTAA			

### 2.5 Statistical analysis

Exact binomial 95% confidence intervals (CIs) were established for the total proportions of infection found herein. The exact Fisher’s test was used to assess statistical differences in infection rates between animal species and collection year; *p*-values less than 0.05 were considered statistically significant. Odds ratio (*OR*) values were calculated to assess the risk of infection according to animal species. Statistical analyses were performed using the online software Epitools - Epidemiological Calculators (20).

## 3 Results

A total number of 911 animals were collected and examined in this study, including 449 dogs and 462 cats. Out of 911 animals, 451 (i.e., 49.5, 95% CI: 46.3–52.7) tested positive for *E. coli,* including 252 (56.1%) that were dogs and 199 (43.1%) that were cats, respectively. The higher prevalence of dogs found herein was statistically significant (*p* < 0.01), combined with a higher risk of infection (*OR* = 1.69). There was no significant statistical difference in prevalence across the years (*p* = 0.150) (Table 2).

**Table 2:** Positivity of dogs and cats to *Escherichia coli* infection and related pathogenic factors and toxins in this study.

Year	*Escherichia coli* Positive/Total (%)				Pathogenic factors (CNF, CDT, LT, ST)			Toxins (vtx1, vtx2, eae)		
	Dog	Cat	Total	95% CI*	Dog	Cat	Tot	Dog	Cat	Total
2017	60/105 (57.1)	10/28 (35.7)	70/133 (52.6)	44.2–60.1	13	1	14	4	0	4
2018	32/50 (64.0)	27/54 (50.0)	59/104 (56.7)	47.1–65.8	1	1	2	4	5	9
2019	33/53 (62.3)	22/54 (40.7)	55/107 (51.4)	42.0–60.7	6	6	12	0	2	2
2020	28/55 (50.9)	22/73 (30.1)	50/128 (39.0)	31.0–47.7	1	2	3	2	3	5
2021	34/54 (63.0)	51/108 (47.2)	85/162 (52.5)	44.8–60.0	1	3	4	2	4	6
2022	34/72 (47.2)	42/90 (46.7)	76/162 (46.9)	39.4–54.6	4	4	8	3	9	12
2023	31/60 (51.6)	25/55 (40.0)	56/115 (48.7)	39.8–57.7	5	8	13	3	4	7
Total	252/449 (56.1)	199/462 (43.1)	451/911 (49.5)	46.3–52.7	31	25	56	18	27	45

*95% CI: 95% confidence interval calculated on the total prevalence.

At least one virulence factor was detected in 22% of the animals (i.e., 12.4% for pathogenicity factors CNF, CDT, LT, ST, and 10.0% for virulence genes vtx1, vtx2, and eae). For CNF, CDT, LT, and ST, there was no significant difference between dogs and cats found (*p* = 1.680); however, cats were significantly more susceptible to verocytotoxins than dogs (*p* = 0.020, *OR* = 2.04).

Out of 252 dogs and 199 cats positive for *E. coli* infections, 31 dogs (i.e., 12.3, 95% CI: 8.8–16.9) and 25 cats (12.6, 95% CI: 8.7–17.9) had shown positivity for at least one toxin (CNF, CDT, LT, and ST); regarding verocytotoxins (vtx1, vtx2, eae), 18 dogs (7.1, 95% CI: 4.6–11.0) and 27 cats (i.e., 13.6, 95% CI: 9.5–19.0).

## 4 Discussion

The high prevalence of *E. coli* in dogs and cats in this study indicates a wide circulation of the infection in both owned and stray animals in Southern Italy, underlying the importance of routine screening for this bacterium to guarantee animal welfare and public health. The screening could be relevant mainly in rural/peri-urban areas with abundant phenomena of free-ranging animals, where stray dogs and cats may overlap with livestock and other companion animals. Indeed, dogs and cats remain under-diagnosed, and given their close proximity to other animals and humans in urban/peri-urban areas, the transmission of the microorganism is expected (1). In this regard, Ahmed et al. (21) highlights the role of companion animals in the spread of STEC strains in the environment. Contacts between animals and their related owners may facilitate the transmission of these strains to humans, as *E. coli* can transfer virulence genes *via* a horizontal transmission pathway (22, 23). For instance, a recent case of haemolytic uremic syndrome has been ascertained in a child who lived with a cat that tested positive for verocytotoxin-producing *E. coli* but showed no signs/symptoms, indicating that this pet was a reservoir of infection for humans (24). Dogs had a higher prevalence of *E. coli* than cats (56.1% *vs* 43.1%, *p* < 0.01), suggesting potentially important differences in the role each species plays as a carrier/reservoir of bacteria that require further investigations.

The absence of a significant statistical difference in prevalence through the years (*p* = 0.150) suggests that the stable circulation of *E. coli* in the study area.

Regarding toxin-producing *E. coli*, very few is known in the literature about dogs and cats (25). The present study highlights a high percentage (i.e., 22%) of animals harboring toxin-producing *E. coli*, indicating the potential role of companion animals in the spread of these zoonotic agents. The risk of infection should be considered, especially when dogs and cats share the same environment with children and immunosuppressed individuals who are more susceptible to contracting the disease in its severe form (26).

The high prevalence of both pathogenicity factor genes (12.4%) and virulence genes (10.0%) is particularly noteworthy as these genes are linked to significant gastrointestinal diseases in humans (5, 6), such as hemorrhagic colitis and haemolytic-uremic syndrome (6).

In particular, the positivity for *E. coli* toxins (CNF, CDT, LT, and ST) occurs similarly in dogs (12.3%) and cats (12.6%), whereas verocytotoxins (vtx1, vtx2, and eae) are found in these animals with different prevalence values (7.1% dogs and 13.6% cats). These findings suggest that whereas dogs have a higher prevalence of *E. coli* infection, cats have a higher incidence of virulence factors/verocytotoxins. These findings are supported by studies in the literature, which show that the prevalence of toxin-producing *E. coli* in dogs ranges from 2.9 to 12.3% in dogs and 13.8 to 23.1% in cats (25).

The findings that cats are significantly more suitable for verocytotoxin production than dogs (*p* = 0.020, *OR* = 2.04) suggest that these felids may act as the main reservoir for toxin-producing *E. coli* (27). However, this hypothesis deserves insights focused on the ecological factors of cats (e.g., dietary habits, environmental exposure, and gut microbiota) that may be involved in the prevalence between cats and dogs (28).

In this context, multicentre studies examining seasonal fluctuations in prevalence or exploring regional differences across broader geographic areas would provide more comprehensive insights.

Finally, our data suggest that epidemiological investigations should be routinized with a holistic approach considering the role of pets such as dogs and cats in the multiple routes of transmission, contamination, and infection by *E. coli*.

## Data Availability

The raw data supporting the conclusions of this article will be made available by the authors, without undue reservation.
